# Birth of a poly(A) tail: mechanisms and control of mRNA polyadenylation

**DOI:** 10.1002/2211-5463.13528

**Published:** 2022-12-07

**Authors:** Juan B. Rodríguez‐Molina, Matti Turtola

**Affiliations:** ^1^ MRC Laboratory of Molecular Biology Cambridge UK; ^2^ Department of Life Technologies University of Turku Finland

**Keywords:** cleavage and polyadenylation factor, cleavage factor, mRNA 3'‐end processing, poly(A) binding protein, poly(A) tail length, polyadenylation

## Abstract

During their synthesis in the cell nucleus, most eukaryotic mRNAs undergo a two‐step 3′‐end processing reaction in which the pre‐mRNA is cleaved and released from the transcribing RNA polymerase II and a polyadenosine (poly(A)) tail is added to the newly formed 3′‐end. These biochemical reactions might appear simple at first sight (endonucleolytic RNA cleavage and synthesis of a homopolymeric tail), but their catalysis requires a multi‐faceted enzymatic machinery, the cleavage and polyadenylation complex (CPAC), which is composed of more than 20 individual protein subunits. The activity of CPAC is further orchestrated by Poly(A) Binding Proteins (PABPs), which decorate the poly(A) tail during its synthesis and guide the mRNA through subsequent gene expression steps. Here, we review the structure, molecular mechanism, and regulation of eukaryotic mRNA 3′‐end processing machineries with a focus on the polyadenylation step. We concentrate on the CPAC and PABPs from mammals and the budding yeast, *Saccharomyces cerevisiae*, because these systems are the best‐characterized at present. Comparison of their functions provides valuable insights into the principles of mRNA 3′‐end processing.

## Graphical abstract

Eukaryotic pre‐mRNAs are cleaved and polyadenylated by the cleavage and polyadenylation complex. The polyadenylation activity of this ~ 1 MDa machinery is orchestrated by poly(A) binding proteins, which decorate the poly(A) tail during its synthesis and guide the mature mRNA through subsequent gene expression steps. We review the structure, molecular mechanism, and regulation of mRNA 3′‐end processing machineries, with a focus on the polyadenylation step.

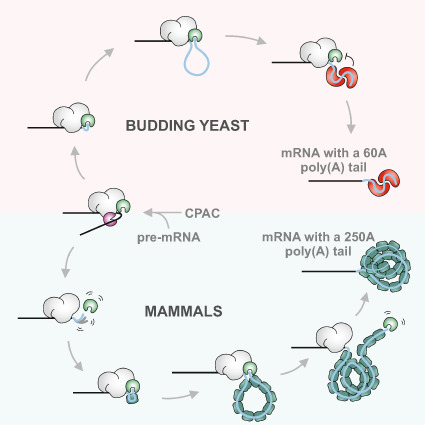

**Abbreviations**
3′‐UTR: 3′‐untranslated region
AMP: adenosine monophosphate
ATP: adenosine triphosphate
CF IIm: mammalian cleavage factor II
CF Im: mammalian cleavage factor I
CF: cleavage factor
CPAC: cleavage and polyadenylation complex
CPF: cleavage and polyadenylation factor
CPSF: cleavage and polyadenylation specificity factor
CstF: cleavage stimulation factor
mCF: mammalian cleavage factor
mPSF: mammalian polymerase specificity factor
mRNA: messenger RNA
mRNP: mRNA ribonucleoprotein particle
PABP: poly(A) binding protein
PAS: polyA signal sequence
poly(A): polyadenosine
pre‐mRNA: precursor mRNA
snRNP: small nuclear ribonucleoprotein

3′‐End processing of eukaryotic mRNAs involves recognition of a polyA signal sequence (PAS) in the nascent pre‐mRNA, followed by endonucleolytic cleavage and polyadenylation of the 5′‐cleavage product (Fig. 1A). These reactions are carried out by a large (~ 1 MDa) and highly conserved multisubunit complex which we will refer to holistically as the cleavage and polyadenylation complex (CPAC). This assembly consists of cleavage factors (CF) together with the cleavage and polyadenylation factor (CPF) in yeast or cleavage and polyadenylation specificity factor (CPSF) in mammals. The CPAC appears to be structurally and enzymatically organized into distinct and highly interconnected modules and subcomplexes (see Box 1) that work together to coordinate 3′‐end processing of mRNAs. Furthermore, the polyadenylation activity of CPAC is intimately controlled by poly(A) binding proteins (PABPs) which decorate the nascent poly(A) tail, instruct the termination of poly(A) tail elongation, and mediate post‐transcriptional gene regulation.

**Fig. 1:**
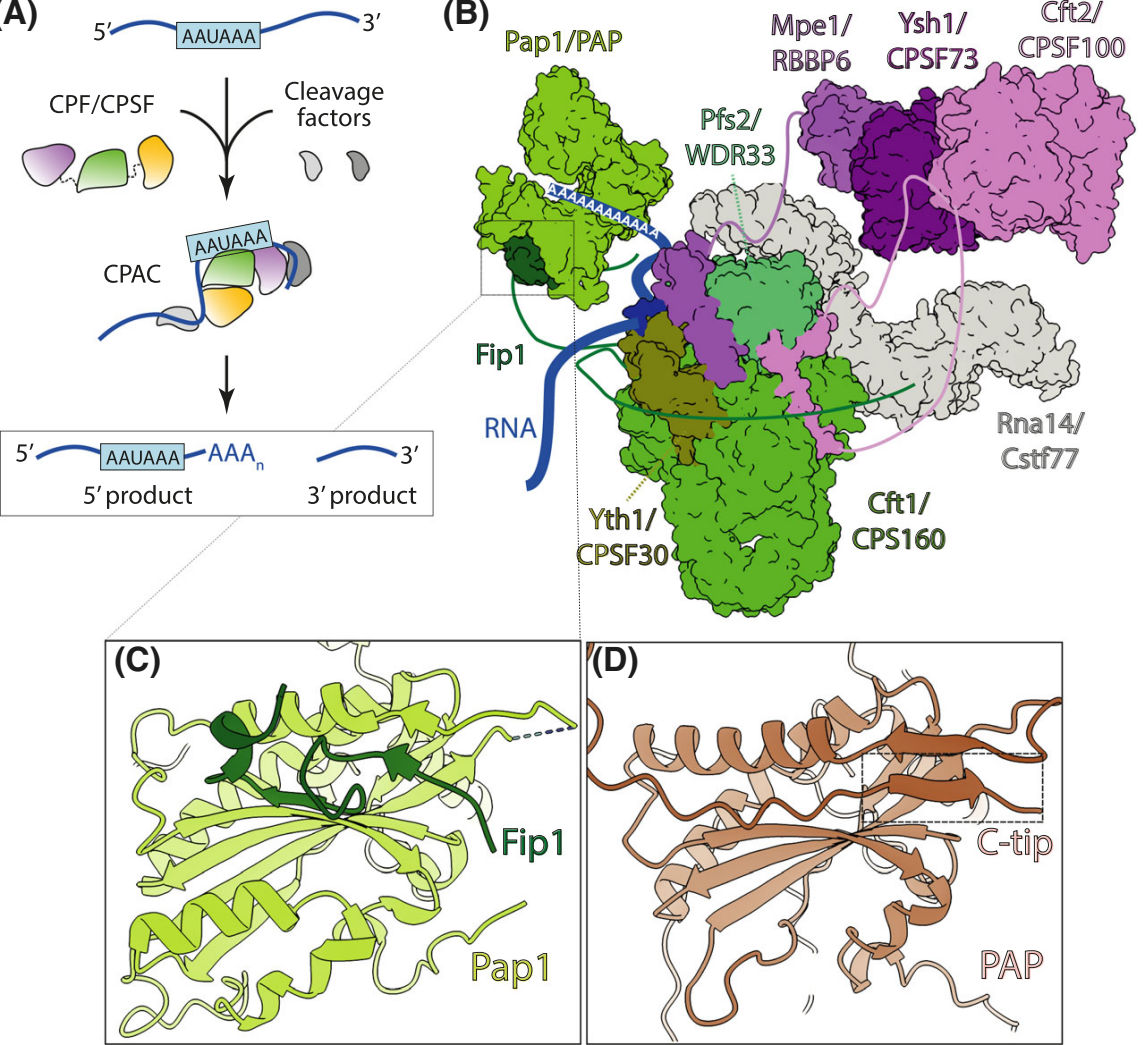
The eukaryotic CPAC. (A) Prior to RNA binding, CPF/CPSF is in a flexible, elongated, and inactive state. Recognition of the polyA signal sequence (A_1_A_2_U_3_A_4_A_5_A_6_) on the RNA by the polymerase module/mPSF (green) and binding of the cleavage factors (gray) to the upstream and downstream sequence elements in the RNA leads to a structural rearrangement that primes the complex for activation [5, 7, 12]. Once in the activation‐competent state, the rearranged CPAC is thought to bring the nuclease module (purple) in close proximity to the cleavage site allowing cleavage and subsequent polyadenylation. The phosphatase module (yellow) is not strictly required for 3′‐end processing but may coordinate the abovementioned transitions. (B) Surface representation of a composite model of the polymerase (shades of green) and nuclease modules (shades of purple) of the yeast CPAC. The corresponding homologous subunits in mammals are also labeled. The HAT domains of Rna14/Cstf77 dimer are also shown in gray. The polyadenylated RNA substrate bound to the Yth1/CPSF30 and Pfs2/WDR33 subunits is depicted in blue. The yeast polymerase module (PDB: 7ZGR) was aligned to the human mPSF‐CstF77 complex (PDB: 6URO). Pap1 is shown as being flexibly tethered by one of the two copies of Fip1 (PDB: 3C66), whereas the second copy is contacting the HAT domain of Rna14/Cstf77. The Ysh1/CPSF73 (PDB: 6I1D) and Cft2/CPSF100 (PDB: 2I7X) subunits of the nuclease module/mCF are flexibly tethered by Cft2/CPSF100 and Mpe1/RBBP6. (C) Cartoon representation of the yeast Pap1‐Fip1 interface. (D) AlphaFold prediction of the human PAP showing the corresponding region shown in (C) to depict the potential binding of the C‐tip regulatory region.

Box 1 **Functional composition of the CPAC**

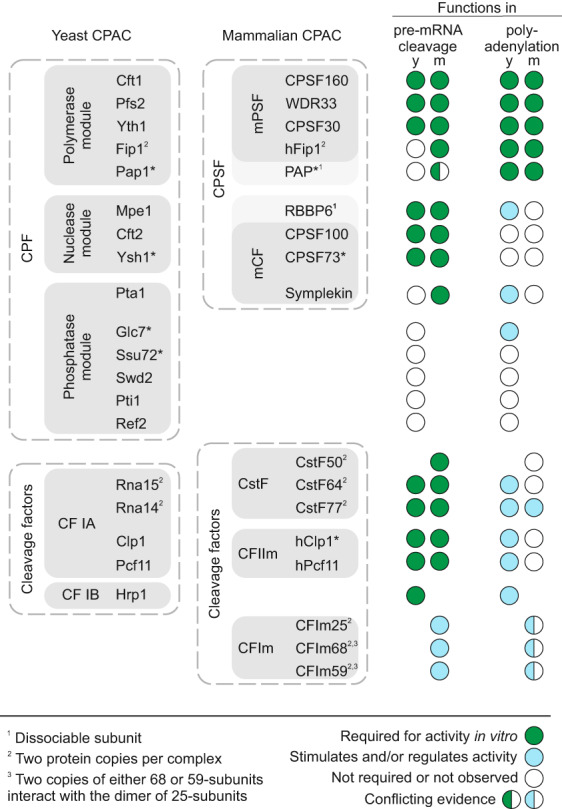

CPF/CPSF and cleavage factors are highlighted by dashed lines. Sub‐complexes (or modules) are indicated by darker shading. Subunits homologous in yeast and mammals are shown side by side. Subunits with enzymatic activity are marked by an asterisk. The requirement of individual yeast (y) and mammalian (m) subunits for the pre‐mRNA cleavage and polyadenylation phases is shown on the right (see the color key).

Here we walk through the nuclear birth of the mRNA poly(A) tail which emerges as a complex and highly regulated process. We highlight the recent progress on the structural and functional characterization of the CPAC of yeast and mammals, and trace how variations in the conserved polyadenylation machineries and their interplay with PABPs result in major differences in polyadenylation in these organisms.

## Recognition of the polyadenylation signal triggers RNA cleavage

The binding of CPAC to the PAS (A_1_A_2_U_3_A_4_A_5_A_6_) on the nascent pre‐mRNA triggers the enzymatic activities of CPAC and defines the 3′‐end of the mRNA. The recognition of PAS is carried out by the five‐zinc finger subunit of the CPAC, Yth1/CPSF30, and the WD40 protein Pfs2/WDR33, in complex with the scaffolding three‐beta propeller protein, Cft1/CPSF160. Together, CPSF160‐WDR33‐CPSF30 along with hFip1 constitute the mammalian polymerase specificity factor (mPSF) (Box 1 and Fig. 1B) [1, 2]. In yeast, the equivalent Cft1‐Pfs2‐Yth1‐Fip1 sub‐complex additionally contains the poly(A) polymerase Pap1 as a constitutive subunit (i.e. Pap1 is always stably bound to the complex) to form the polymerase module [3]. mPSF and the polymerase module function as a rigid scaffold to coordinate 3′‐end processing of mRNAs.

In yeast and humans, Yth1/CPSF30 binds the first two nucleotides (A_1_A_2_) of the PAS. Here, the zinc finger 2 makes highly conserved base‐specific contacts. In humans, CPSF30 makes additional base‐specific contacts with the A_4_A_5_ of the PAS via its zinc finger 3, and a non‐conserved N‐terminal region in WDR33 specifically stabilizes a Hoogsteen base pair between U_3_ and A_6_ [2, 4, 5, 6]. This WDR33‐RNA interaction may explain the higher affinity of the human CPAC for the PAS sequence compared to the yeast complex. Pfs2/WDR33 may also contribute to RNA binding via additional charged surfaces on its flat solvent‐exposed side, but whether this contributes in a sequence‐specific manner is unclear [3].

PolyA signal sequence recognition then triggers activation of the CPAC endonuclease, Ysh1/CPSF73, which cleaves the pre‐mRNA ~ 20 nucleotides downstream of the PAS. The endonuclease is part of a multisubunit subcomplex known as mammalian Cleavage Factor (mCF) and yeast nuclease module [3, 7]. The composition of these sub‐complexes and their requirement in CPAC cleavage activity are summarized in Box 1. It should be emphasized that the activation of the endonuclease additionally requires cleavage factors, as described later. Ysh1/CPSF73 interacts with a related pseudonuclease, Cft2/CPSF100 and the scaffolding protein Pta1/Symplekin [8, 9]. Recent studies show that Mpe1/RBBP6 is universally required for correct activation of Ysh1/CPSF73 likely through a conserved mechanism of action [6, 7, 10, 11]. Importantly, although Mpe1 shows a strong interaction with Ysh1 [12] and is a constitutive subunit of the yeast CPAC, RBBP6 has weaker affinity for CPSF73 and is therefore not a constitutive subunit of the human CPAC [7, 10, 13]. Recruitment of RBBP6 to the mammalian CPAC may require recognition of the PAS sequence, stable RNA binding, or conformational changes which could signal a quality control checkpoint before committing the complex to pre‐mRNA cleavage.

Pta1/Symplekin is a scaffolding protein which bridges the three enzymatic activities of the CPAC (polymerase, endonuclease, and phosphatases, discussed below) and may be an important node to integrate and relay regulatory signals between the enzymatic components. Pta1 directly interacts with the N‐terminal domain of poly(A) polymerase Pap1 [14], and Pta1/Symplekin form conserved interactions with Cft2/CPSF100 and the endonuclease Ysh1/CPSF73 [8, 15]. Curiously, Pta1 and Symplekin are part of distinct subcomplexes in yeast and mammals (Box 1). In mammals, Symplekin belongs to the mCF and is required for cleavage activity. In yeast, Pta1 is part of the phosphatase module which couples pre‐mRNA 3′‐end processing to transcription termination by dephosphorylating the C‐terminal domain of RNA polymerase II [16]. However, the phosphatase module is dispensable for cleavage and polyadenylation activities of the yeast CPAC. To date, an equivalent phosphatase module has not been identified in mammals, but the conserved human Ssu72 phosphatase does interact with Symplekin [17]. Additional subunits of the yeast phosphatase module have identifiable human homologs: Swd2/WDR82, Glc7/PP2A, and potentially Ref2/PNUTS. Recent studies have begun to elucidate a role for these proteins in 3′‐end processing [13, 18, 19].

Cleavage factors play essential roles in activating the endonuclease (Fig. 1A). In yeast the multi‐subunit complex CF IA (composed of Rna14, Rna15, Clp1, and Pcf11), and the single subunit CF IB/Hrp1 are required for correct activation of the CPF endonuclease, Ysh1 [12, 20, 21, 22]. These factors bind sequence elements upstream and downstream the PAS that are important for their role in regulating 3′‐end processing [23, 24, 25, 26]. CF IA and CF IB also stimulate and control polyadenylation activity of the CPAC, as discussed later. In mammals, the functionally equivalent CF IA homologs are split between two stable complexes: cleavage stimulation factor (CstF, consisting of Cstf77, Cstf64, and Cstf50 subunits) [27, 28, 29] and the mammalian cleavage factor II (CF IIm, consisting of Pcf11 and Clp1) [30, 31] (Box 1). The mammalian cleavage factor I (CF Im) is not required for activation of cleavage [7, 10], but does bind sequence motifs upstream of the PAS to stabilize assembly of the CPAC on its substrate and regulate cleavage site selection [32].

## From cleavage to polyadenylation

Cleavage of the pre‐mRNA generates a 5′‐product and a 3′‐product. The 5′‐product of the cleaved pre‐mRNA is polyadenylated by the poly(A) polymerase (Pap1 in yeast, PAP in vertebrates). The 3′‐product, which is still bound to transcribing RNA polymerase II, is degraded by the torpedo exonuclease, Rat1/XRN2. The exonuclease activity of Rat1/XRN2 on the 3′‐cleavage product ultimately leads to transcription termination by dislodging the RNA Polymerase II from the DNA [33, 34]. The 3′‐cleavage product, which is downstream of the cleavage site, contains sequence elements bound by CF IA or CstF which were necessary for activating CPAC cleavage activities [12]. Thus, it is reasonable to speculate that digestion of the 3′‐product by Rat1/XRN2 effectively enforces the directionality of 3′‐end processing by limiting re‐cleavage of the RNA substrate, thereby promoting productive polyadenylation.

Pap1/PAP is flexibly tethered to the complex through its interaction with Fip1/hFip1, which should facilitate easy access to the 5′‐product following cleavage [35, 36] (Fig. 1B). Whereas human PAP is weakly bound to hFip1 and is not a constitutive component of the mammalian CPAC [1, 13], yeast Pap1 is a constitutive subunit of the CPAC. Indeed, a non‐conserved region of yeast Fip1 appears to strongly bind a conserved cleft in Pap1 [37] (Fig. 1C), but in humans multiple low‐affinity regions in hFip1 are required for its interaction with PAP [38]. The differential affinities of Pap1/PAP within CPAC complexes may underlie critical species‐specific regulatory mechanisms of polyadenylation, as discussed later in this review.

The stoichiometry of Fip1‐Pap1/PAP within the CPAC has been the subject of recent structural studies which agree that two copies of the Fip1 subunit are able to interact with Yth1/CPSF30 (Fig. 1B). Specifically, crystal structures and solution nuclear magnetic resonance studies reveal extensive high‐affinity interaction of one Fip1 molecule with zinc finger 4 of Yth1/CPSF30, and another Fip1 molecule interacting with zinc finger 5 albeit with lower affinity [36, 38, 39]. This would, in principle, allow up to two polymerase molecules to be accommodated within the CPAC, which has been observed using native mass spectrometry analysis [3]. Further studies are required to address the functional relevance and regulatory potential of differential Fip1‐PAP stoichiometries on polyadenylation and gene expression.

There may be CPAC states that favor cleavage or polyadenylation depending on the conformation of the substrate RNA, the recruitment of cleavage factors and non‐constitutive subunits, and the integration of regulatory signals. hFip1, for example, has been suggested to mediate some of these transitions. Specifically, the Cstf77 homodimer within CstF binds to the N‐terminal region of two copies of hFip1 (Fig. 1B). This interaction is mutually exclusive with the interaction between hFip1 and PAP [38] and may explain why PAP is not part of the pre‐cleavage complex [13]. Presumably, this state of the CPAC (without PAP) is competent for (and would even promote) cleavage, but recent studies show opposing views on the requirement for PAP in the cleavage activity of the CPAC [7, 10]. Clarifying the role of PAP in cleavage will require further studies. It is clear, however, that Cstf77 can prevent PAP incorporation into the CPAC, thereby inhibiting polyadenylation [38]. This may offer clues as to how the CPAC transitions from cleavage to polyadenylation. Of note, the yeast orthologue of Cstf77, Rna14 has been reported to interact with Fip1 as well [40], but the functional significance of this connection is unknown.

The transition from cleavage to polyadenylation may be regulated by Pta1 and the phosphatase activity of the CPAC, although the phosphatase module is not strictly required for mRNA 3′‐end processing. Phosphorylation of Pta1 inhibits polyadenylation activity presumably by stabilizing its binding to Pap1 [14, 41]. This inhibition is relieved by the Glc7 phosphatase [41], suggesting that Glc7 is an important regulator of CPAC states aiding in its transition from cleavage to polyadenylation‐competent states. Importantly, the regulation of this transition could allow the choice between abortive or productive synthesis of mature mRNA. We can speculate that unadenylated or short‐tailed mRNAs observed in certain conditions [42, 43] represent a stalled state of CPAC which did not fully commit to polyadenylation following RNA cleavage. An unadenylated 5′‐cleavage product is prominent in some experiments assaying coupled cleavage and polyadenylation [3, 10, 12], whereas in other similar experiments this intermediate does not accumulate [7, 43]. This indicates that the efficiency of this transition is sensitive to experimental conditions *in vitro*.

Elucidating the conformational changes between the functional states of the CPAC will reveal how the RNA is delivered to the polymerase following cleavage, the role cleavage factors play in coordinating these transitions and how the complex is ultimately recycled to act on a new RNA substrate.

## Poly(A) tail elongation

The CPAC polymerase uses the cleaved pre‐mRNA 3′‐end as a primer to start synthesizing the poly(A) tail. Within the U‐shaped Pap1/PAP, a large cleft is formed between the palm and C‐terminal domains which encloses three nucleotides of RNA and harbors the enzyme active site on one side within the fingers and palm domains (Fig. 1B) [44, 45, 46]. In each nucleotide addition cycle, ATP binding into the active site induces cleft closure and the catalysis of an AMP incorporation into the RNA 3′‐end. Upon nucleotide addition, the cleft then opens to allow the release of the products; pyrophosphate and the extended poly(A) tail [44, 47]. As a result of limited binding to the RNA primer, the polymerizing activity of Pap1/PAP is distributive. In other words, the enzyme adds only one or few nucleotides before dissociating from its RNA substrate [44, 48, 49, 50].

Because the rate of polyadenylation is limited by the stability of polymerase binding to the RNA 3′‐end, the specificity and processivity of polyadenylation are controlled by factors that modulate this interaction. CPAC binding to the PAS, as detailed above, links the pre‐mRNA and polymerase together. This tethering of substrate and enzyme increases the rate of poly(A) tail addition but becomes progressively weaker when the poly(A) tail grows longer [51, 52, 53]. Additional contacts to the RNA sequences upstream of the PAS may be required to enhance pre‐mRNA binding to the CPAC. For example, cleavage factors IA and IB in yeast may stimulate and control poly(A) tail elongation by mediating such contacts [3, 20, 21, 43, 54]. In mammals, CF Im has been proposed to play a similar role in stimulating polyadenylation [55, 56], but this activity could not be recapitulated in a reconstituted system [7]. Further research is needed to elucidate how cleavage factors modulate polyadenylation and whether their differential recruitment could provide a means for regulating poly(A) tail synthesis in a gene or transcript isoform‐specific manner.

**Fig. 2:**
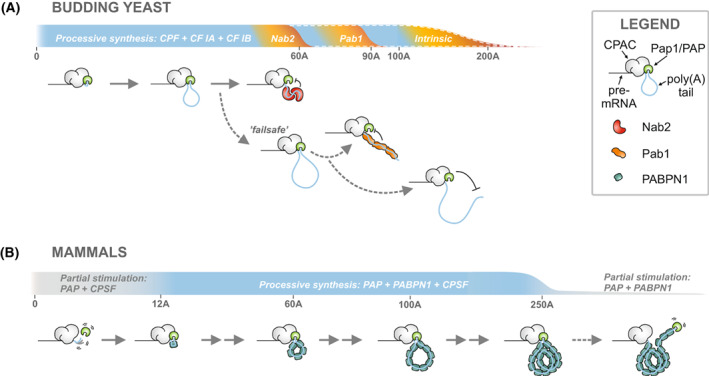
Different mechanisms of mRNA poly(A) tail elongation and length control in the budding yeast and mammals. The main phases of mRNA poly(A) tail elongation and termination are shown as cartoons (see Legend) below the horizontal bars which provide the coordinates for poly(A) tail length and the relative rates of polyadenylation at each phase (indicated by color; blue = high, gray = intermediate, yellow/orange = low). The downward slopes depict termination of poly(A) tail synthesis. (A) The budding yeast CPAC polyadenylates the pre‐mRNA with high basal activity. Polyadenylation is terminated primarily by Nab2 after the synthesis of ~ 60 adenosines. If Nab2 is not available, then uncontrolled polyadenylation is prevented through fail‐safe termination pathways (dashed arrows and coordinate bar) by Pab1 or by the intrinsic mechanism of CPAC which restrict poly(A) tail lengths to ~ 90 and 100–200 adenosines, respectively. Nab2 and Pab1 are hypothesized to “measure” the poly(A) tail lengths by forming oligomeric structures that prevent Pap1 accessing the RNA 3′‐end. The high basal polyadenylation activity as well as the intrinsic length control require cleavage factors CF IA and CF IB within the CPAC (model adapted from [43]). (B) Mammalian CPAC has low basal polyadenylation activity due to weak association of PAP with the CPSF and poly(A) tail. After a slow addition of 10–12 adenosines, binding of first PABPN1 to the nascent tail stabilizes PAP's association with the poly(A) tail. Now PABPN1 and CPSF cooperatively stimulate PAP's activity allowing rapid poly(A) tail elongation. In the course of elongation, PABPN1 oligomerizes on the growing poly(A) tail and ultimately forms a size‐limited spherical structure with a ~ 250‐adenosine tail. Although PABPN1 continues to stimulate polyadenylation beyond this point, the spherical structure prevents the CPSF from stimulating PAP's activity, which terminates the processive phase of elongation (model adapted from [52]).

Despite the high degree of conservation between the yeast and human CPACs, poly(A) tails are elongated in a very different manner in these organisms. The difference can be attributed, at least to some extent, to the details of how Pap1/PAP is tethered to the rest of the CPAC (discussed above) and brought into the proximity of the RNA 3′‐end. In yeast, the stable Pap1‐Fip1 interaction provides sufficient processivity for the fully assembled CPAC to elongate poly(A) tails rapidly to 100–200 adenosines [3, 6, 43] (Fig. 2A). It is also possible that an RNA‐binding site within the C‐terminal domain of Pap1 [45, 57, 58] contributes to orienting and maintaining the poly(A) tail in a conformation conducive for processive polyadenylation.

On the other hand, PAP is not a stable subunit of the mammalian CPAC [1, 13]. Although hFip1 links PAP to the CPAC and U‐rich regions of the pre‐mRNA [38, 59], this connection provides only partial polyadenylation activity. Mammalian CPAC reaches full activity in the presence of the nuclear PABP, PABPN1, which binds PAP and the nascent poly(A) tail, and increases the apparent affinity of PAP for the poly(A) tail [51, 52, 60]. Individually either CPSF or PABPN1 can stimulate PAP's activity by 50‐fold, but together, they increase the polyadenylation rate by 15 000‐fold [53, 61]. As a result of this cooperative action polyadenylation proceeds in a biphasic manner (Fig. 2B): The first 10–12 adenosines which do not allow simultaneous binding of PAP and PABPN1 are added slowly whereas the subsequent elongation up to 250 adenosines proceeds rapidly supported by sequential binding of PABPN1 molecules to the nascent poly(A) tail [52].

Notably, as opposed to the human system, in the budding and fission yeasts PABPs do not appear to be required during the elongation phase [42, 43, 62, 63], instead, they inhibit the activity of Pap1 and act to terminate polyadenylation in a timely manner (see the next section). Nevertheless, the association of PABPs may prevent the CPAC from re‐cleaving its polyadenylated pre‐mRNA substrate at the same site [64], thereby providing additional directionality to the 3′‐end processing steps.

## Termination of polyadenylation and the control of poly(A) tail length

Poly(A) tails on newly synthesized mRNAs are uniform in length implying that all mRNAs are polyadenylated in an identical manner within an organism [43, 63, 65]. The intriguing biochemical question of such “length control” is how to stop the untemplated polyadenylation reaction at the right moment? The polyadenylation activity of CPAC is controlled by PABPs that associate with the nascent poly(A) tails and instruct termination of poly(A) tail synthesis. Interestingly, the lengths of poly(A) tails on yeast and mammalian mRNAs are strikingly different (60 and 250 adenosines, respectively) due to distinct termination mechanisms which reflect the different modes of poly(A) tail elongation (discussed in the previous chapter) and the acquisition of non‐homologous PABPs for controlling this process (Fig. 2).

In yeast, the otherwise efficient poly(A) tail elongation is inhibited by the association of Nab2, a CCCH‐type zinc finger protein, resulting in 60‐adenosine long poly(A) tails [43, 64, 66, 67, 68]. If Nab2 is not available, polyadenylation can be terminated at 90 adenosines by Pab1, a PABP containing four RRM‐domains [42, 43, 64, 66]. Importantly, Pab1 localizes mostly in the cytoplasm but shuttles to the nucleus [69]. In contrast, mammalian mRNA poly(A) lengths are controlled by PABPN1, a single RRM‐domain‐containing protein, which provides means for both stimulating poly(A) tail elongation and terminating it once the poly(A) tail is ~ 250 adenosines long [51, 52, 53, 60, 63].

A general feature of PABP‐mediated length control is the multimerization of proteins on the nascent poly(A) tail. How these proteins “measure” the exact poly(A) tail length and terminate polyadenylation is best characterized for PABPN1. As discussed before, PABPN1 stimulates polyadenylation by increasing the apparent affinity of PAP for the poly(A) [60]. Successive association of PABPN1 molecules to the growing tail forms an oligomeric filament, in which individual molecules cover ~ 11 nucleotides and the binding displays moderate cooperativity. The filament collapses into a spherical particle which can grow up to 20 nm wide accommodating ~ 15–20 PABPN1 molecules bound to a ~ 250‐adenosine tail. This is a size limit that prevents more PABPN1 from associating within the particle [70, 71, 72] (Fig. 2B). Further extension of the tail beyond the length contained in the particle is no longer compatible with the interaction between PAP and the rest of the CPAC. This breaks the tripartite connection, which is required for the cooperative stimulation of polyadenylation, and thereby terminates processive synthesis [52]. Nab2 and Pab1 operate on poly(A) length control with a different mechanism as their binding to the poly(A) tail inhibits Pap1 activity [43, 64, 66] (Fig. 2A). Nab2 interacts with poly(A) RNA in an unusual way: two Nab2 molecules bring three of their seven zinc‐fingers [73] together to create a binding site for a stretch of 11 adenosines [74]. However, it remains unclear how Nab2 measures the length of a full‐sized 60‐adenosine tail and whether such dimerization mediates termination of polyadenylation [68]. On the other hand, the 30‐nucleotide footprint of Pab1 directly suggests that the association of three Pab1 molecules on the poly(A) tail [75, 76] is responsible for inhibiting polymerization after the synthesis of a 90‐adenosine tail [43].

We speculate that the evolution of PABPN1‐stimulated polyadenylation in animal cells, and presumably in plants, may have enabled the robust synthesis of  longer poly(A) tails compared to those in fungi [77, 78] which appear to lack such mode of regulation [62, 63, 79]. In this scenario, the early termination by Nab2 or Pab1‐type PABPs, still utilized by the present‐day fungi, became circumvented by the continuous loading of PABPN1 molecules during elongation. That being said, animal orthologues of Nab2 (ZC3H14) and Pab1 (PABPC1‐4) have been suggested to regulate nuclear polyadenylation [73, 80, 81]. How these, and other proteins such as nucleophosmin [82], function in relation to the PABPN1 pathway awaits further studies.

Recently, a functional characterization of the yeast CPAC reconstituted from recombinant proteins uncovered a surprising new mechanism for controlling poly(A) length which is independent of PABPs and inherent to the CPAC machinery. This “intrinsic length control” requires the cleavage factors CF IA and CF IB, and terminates polyadenylation after the addition of 100–200 adenosines [43]. The mechanism for intrinsic length control remains unclear, but could involve steric occlusion or sequestration of the poly(A) tail by the RNA‐binding surfaces of CPAC, or product inhibition of Pap1 by the long poly(A) tail. Nevertheless, termination of polyadenylation by the intrinsic activity of CPAC, as well as by Pab1, provides fail‐safe systems in conditions when the primary Nab2 pathway is unable to control all polyadenylation events (Fig. 2A). This may prevent excessive polyadenylation and enable recycling of CPAC, thereby increasing the robustness of gene expression.

Finally, because PABPs stay bound to the poly(A) tail after termination, length control is an integral part of the assembly of the mRNA ribonucleoprotein particle (mRNP) [83]. The nuclear availability of PABPs, which changes in different conditions [80, 84, 85, 86, 87, 88, 89], will impact the pathway utilized for poly(A) length control and thus the composition and stoichiometry of mRNP‐bound PABPs. Therefore, termination of polyadenylation links mRNA 3′‐end processing to the subsequent steps in gene expression: mRNP release from chromatin, nuclear export, remodeling after passage through the nuclear pore complex, translation [65, 90, 91, 92, 93, 94], and mRNA degradation, as detailed later in the review.

## Polyadenylation as a gene regulatory step

Considering the central role of mRNA polyadenylation in gene expression, it is no surprise that the CPAC machineries are regulated by a multitude of accessory factors and signaling pathways. Much research has focused on how choices of alternative pre‐mRNA cleavage sites control early transcription termination [95, 96, 97] and generate different transcript 3′‐UTR (3′‐untranslated region) isoforms [98, 99, 100]. Nonetheless, a number of studies also illustrate polyadenylation as a gene regulatory step. Pap1/PAP, other subunits of CPAC and PABPs, can be post‐translationally modified or bound by regulatory proteins to control mRNA expression through polyadenylation [14, 41, 82, 85, 101, 102, 103, 104, 105, 106, 107]. Furthermore, mammalian CPAC is regulated by components of the splicing machinery [108, 109, 110, 111, 112, 113] in order to coordinate splicing with pre‐mRNA 3′‐end processing [114, 115, 116]. Here, the regulation of PAP by the U1 small nuclear ribonucleoprotein (snRNP) and U2AF 65, which help to define the 5′ and 3′‐splice sites, respectively, may be relevant. Conversely, splicing of a subset of terminal introns can be stimulated by PAP and the poly(A) tail‐bound PABPN1 [117, 118].

Much of the PAP regulation in mammals is wired through its C‐terminal 240 residues which appear to be mostly unstructured. For example, U2AF 65, as well as the U1 snRNP subunits U1 70K and U1A, all employ similar motifs to inhibit the activity of PAP by binding to a region that spans the last 20 C‐terminal amino acids of PAP (referred here as C‐tip) [109, 110, 111]. C‐tip seems to be important for PAP's processivity but is not present in the current PAP structures. High conservation of C‐tip sequence between human, bovine, and *Xenopus* PAPs [109] further suggests that the evolution of this part of the protein is limited by functional constraints.

In an attempt to understand mechanistically how such regulation might work, we examined the AlphaFold predicted structures of several vertebrate PAPs [119, 120]. Curiously, in these models the C‐tip folds back to interact with the C‐terminal domain of PAP, and this putative site of interaction overlaps with a corresponding region in Pap1 that binds yFip1 (Fig. 1C,D). An important question is whether the binding of regulatory factors to the C‐tip could tune the strength of Fip1‐PAP interaction and affect the recruitment of PAP to the CPAC. In one scenario, regulator binding could displace C‐tip from the surface of PAP, thereby permitting hFip1 binding to PAP in a manner observed in yeast. Here regulator binding would promote the recruitment of PAP to the CPAC, which appears at odds with the observed inhibition. In an alternative scenario, a regulator could interact with the C‐tip bound to the surface of PAP, as in Fig. 1D, and as a result sterically occlude hFip1 binding. This would inhibit the recruitment of PAP to the CPAC. Testing mechanistic questions and predictions like these is now possible with the recently reconstituted recombinant polyadenylation systems [2, 10, 38].

## Birth and demise: a brief view on poly(A) tail dynamics during mRNA lifecycle

Polyadenylation is a constitutive and essential step in gene expression because unadenylated pre‐mRNAs are exported slowly and degraded by nuclear RNA surveillance factors [121, 122]. The only mRNAs lacking poly(A) tails are the metazoan replication‐dependent histone mRNAs which protect their 3′‐ends with a stem‐loop structure and which are processed by the U7 snRNP complex [123]. In all other protein‐coding transcripts, poly(A) tails protect them from nuclear degradation, stimulate translation, and play a central role in their controlled degradation in the cytoplasm [124, 125].

After their initial synthesis in the nucleus, poly(A) tails undergo gradual shortening (deadenylation) catalyzed by RNA deadenylase enzymes. Complete removal of the tail releases poly(A)‐bound PABPs triggering decapping and degradation of the mRNA from both 5′ and 3′‐ends [126, 127, 128]. Indeed, the varying poly(A) tail lengths observed in total mRNA ensembles reflect a mixture of different aged molecules each deadenylated with an mRNA‐, cell type‐ and condition‐specific rate [77, 129, 130, 131]. The variations in deadenylation rates are governed by, for example, the recruitment of deadenylases and their co‐factors, translation efficiency, and RNA localization [125]. A textbook view is that the removal of the poly(A) tail is the rate‐limiting step in mRNA degradation [126], which is supported by recent studies in mouse fibroblasts and yeast [127, 130]. Assuming this model, the defined initial tail length, ensured by the nuclear length control systems, contribute to establishing precise mRNA half‐lives by fixing the number of adenosines that need to be removed before degradation occurs [61]. However, this direct relationship between poly(A) tail lengths and RNA half‐lives is confounded by observations that deadenylation rates vary across the length of the poly(A) tail, and short‐tailed mRNAs display large variation in their degradation rates [125, 130, 131, 132]. Furthermore, these rates can exist within a regime in which RNA degradation is temporally uncoupled from deadenylation [133]. Nevertheless, the corollary of the observed global poly(A) tail length control is that all mRNAs enter the deadenylation process from the same starting point.

Importantly, certain metazoan mRNAs undergo a second round of polyadenylation in the cytoplasm. Such regulation is important, for example, for translational activation of maternal mRNAs in oocytes and early embryos. Cytoplasmic polyadenylation employs some of the same components of the CPAC which are responsible for nuclear mRNA polyadenylation [134]. In addition, a repertoire of non‐canonical poly(A) polymerases and terminal uridylyltransferases can extend poly(A) tails in the nucleus or the cytoplasm, often adding residues other than adenosine in a process named “mixed tailing” in order to impact mRNA stability and translatability [129, 135, 136]. The recent observation of N6‐methylation of adenines within the poly(A) tail adds to the list of ways to slow the activity of deadenylases and stabilize mRNAs [137].

In summary, despite their monotonic sequence, poly(A) tails are highly dynamic structures which integrate various cellular signals to modulate mRNA expression at the post‐transcriptional level.

## Conclusions and future directions

Cleavage and polyadenylation complex machineries of mammals and yeast display overall a high level of structural conservation and share many functional properties. However, a few structural divergences affecting interactions between essential components of CPAC reflect a requirement for specialization in gene expression and have resulted in important functional differences in how mRNA poly(A) tails are synthesized across species. In the future, the study of CPAC and PABPs will have to be extended to other eukaryotes, including plants, protists, and other fungal species. This will help to establish universal rules for 3′‐end processing (if any) and give insights into species‐specific specialization. These exceptions may, for example, inspire solutions to prevent the growth of fungal pathogens, which infect animals and plants, by targeting their fungal‐specific features of poly(A) tail synthesis.

Future mechanistic investigations of RNA 3′‐end processing should pursue to visualize the CPAC in the pre‐cleavage, post‐cleavage, polyadenylating, and termination states. Understanding the transitions and dynamics between these states will be challenging, and will require a combination of structural, biochemical, and biophysical approaches. Furthermore, how PABPs interact with the poly(A) RNA and regulate the activity of CPAC will have to be incorporated into these models. Now that reconstituted systems are in place, it has become possible to address in detail how various regulators of CPAC modulate its activities. Finally, these mechanistic queries will have to be combined with systems‐level approaches to investigate how the regulation of poly(A) tail synthesis is integrated within gene expression pathways. For example, do various nuclear PABPs in mammals or paralogous genes of CPAC subunits in plants [138] impose gene, cell type, or condition‐specific regulation of poly(A) length control and mRNA expression?

## Conflict of interest

The authors declare no conflict of interest.

## Author contributions

MT conceived the review. JBR‐M and MT wrote the review.

## References

[1] Chan SL , Huppertz I , Yao C , Weng L , Moresco JJ , Yates JR 3rd , et al. CPSF30 and Wdr33 directly bind to AAUAAA in mammalian mRNA 3′ processing. Genes Dev. 2014;28:2370–80. 25301780 10.1101/gad.250993.114 PMC4215182

[2] Schönemann L , Kühn U , Martin G , Schäfer P , Gruber AR , Keller W , et al. Reconstitution of CPSF active in polyadenylation: recognition of the polyadenylation signal by WDR33. Genes Dev. 2014;28:2381–93. 25301781 10.1101/gad.250985.114 PMC4215183

[3] Casañal A , Kumar A , Hill CH , Easter AD , Emsley P , Degliesposti G , et al. Architecture of eukaryotic mRNA 3′‐end processing machinery. Science. 2017;358:1056–9. 29074584 10.1126/science.aao6535 PMC5788269

[4] Clerici M , Faini M , Aebersold R , Jinek M . Structural insights into the assembly and polyA signal recognition mechanism of the human CPSF complex. Elife. 2017;6:e33111. 29274231 10.7554/eLife.33111 PMC5760199

[5] Sun Y , Zhang Y , Hamilton K , Manley JL , Shi Y , Walz T , et al. Molecular basis for the recognition of the human AAUAAA polyadenylation signal. Proc Natl Acad Sci USA. 2017;115:E1419–28. 29208711 10.1073/pnas.1718723115 PMC5816196

[6] Rodriguez‐Molina JB , Reilly FJO , Fagarasan H , Skehel JM , Rappsilber J , Passmore LA . Mpe1 senses the binding of pre‐mRNA and controls 3′end processing by CPF. Mol Cell. 2022;82:2490–504.e12. 35584695 10.1016/j.molcel.2022.04.021 PMC9380774

[7] Schmidt M , Kluge F , Sandmeir F , Kühn U , Schäfer P , Tüting C , et al. Reconstitution of 3′ end processing of mammalian pre‐mRNA reveals a central role of RBBP6. Genes Dev. 2022;36:195–209. 35177537 10.1101/gad.349217.121 PMC8887130

[8] Zhang Y , Sun Y , Shi Y , Walz T , Tong L . Structural insights into the human pre‐mRNA 3′‐end processing machinery. Mol Cell. 2020;77:800–9.e6. 31810758 10.1016/j.molcel.2019.11.005 PMC7036032

[9] Mandel CR , Kaneko S , Zhang H , Gebauer D , Vethantham V , Manley JL , et al. Polyadenylation factor CPSF‐73 is the pre‐mRNA 3′‐end‐processing endonuclease. Nature. 2006;444:953–6. 17128255 10.1038/nature05363 PMC3866582

[10] Boreikaite V , Elliott TS , Chin JW , Passmore LA . RBBP6 activates the pre‐mRNA 3′ end processing machinery in humans. Genes Dev. 2022;36:210–24. 35177536 10.1101/gad.349223.121 PMC8887125

[11] Lee SD , Moore CL . Efficient mRNA polyadenylation requires a ubiquitin‐like domain, a zinc knuckle, and a RING finger domain, all contained in the Mpe1 protein. Mol Cell Biol. 2014;34:3955–67. 25135474 10.1128/MCB.00077-14 PMC4386453

[12] Hill CH , Boreikaite V , Kumar A , Casanal A , Girbig M , Skehel M , et al. Activation of the endonuclease that defines mRNA 3′ ends requires incorporation into an 8‐subunit core cleavage and polyadenylation factor complex. Mol Cell. 2019;73:1217–31.e11. 30737185 10.1016/j.molcel.2018.12.023 PMC6436931

[13] Shi Y , Campigli D , Giammartino D , Taylor D , Sarkeshik A , Rice WJ , et al. Molecular architecture of the human pre‐mRNA 3′ processing complex. Mol Cell. 2009;33:365–76. 19217410 10.1016/j.molcel.2008.12.028 PMC2946185

[14] Ezeokonkwo C , Ghazy MA , Zhelkovsky A , Yeh PC , Moore C . Novel interactions at the essential N‐terminus of poly(A) polymerase that could regulate poly(A) addition in *Saccharomyces cerevisiae* . FEBS Lett. 2012;586:1173–8. 22575652 10.1016/j.febslet.2012.03.036 PMC3587332

[15] Ghazy MA , He X , Singh BN , Hampsey M , Moore C . The essential N terminus of the Pta1 scaffold protein is required for snoRNA transcription termination and Ssu72 function but is dispensable for pre‐mRNA 3′‐end processing. Mol Cell Biol. 2009;29:2296–307. 19188448 10.1128/MCB.01514-08 PMC2663318

[16] Cossa G , Parua PK , Eilers M , Fisher RP . Protein phosphatases in the RNAPII transcription cycle: erasers, sculptors, gatekeepers, and potential drug targets. Genes Dev. 2021;35:658–76. 33888562 10.1101/gad.348315.121 PMC8091971

[17] Xiang K , Nagaike T , Xiang S , Kilic T , Beh MM , Manley JL , et al. Crystal structure of the human symplekin‐Ssu72‐CTD phosphopeptide complex. Nature. 2010;467:729–33. 20861839 10.1038/nature09391 PMC3038789

[18] Cortazar MA , Sheridan RM , Erickson B , Fong N , Glover‐Cutter K , Brannan K , et al. Control of RNA pol II speed by PNUTS‐PP1 and Spt5 dephosphorylation facilitates termination by a “sitting duck torpedo” mechanism. Mol Cell. 2019;76:896–908.e4. 31677974 10.1016/j.molcel.2019.09.031 PMC6927536

[19] Lee JH , You J , Dobrota E , Skalnik DG . Identification and characterization of a novel human PP1 phosphatase complex. J Biol Chem. 2010;285:24466–76. 20516061 10.1074/jbc.M110.109801 PMC2915683

[20] Gross S , Moore C . Five subunits are required for reconstitution of the cleavage and polyadenylation activities of *Saccharomyces cerevisiae* cleavage factor I. Proc Natl Acad Sci USA. 2001;98:6080–5. 11344258 10.1073/pnas.101046598 PMC33425

[21] Barnwal RP , Lee SD , Moore C , Varani G . Structural and biochemical analysis of the assembly and function of the yeast pre‐mRNA 3′ end processing complex CF I. Proc Natl Acad Sci USA. 2012;109:21342–7. 23236150 10.1073/pnas.1214102110 PMC3535613

[22] Gordon JMB , Shikov S , Kuehner JN , Liriano M , Lee E , Stafford W , et al. Reconstitution of CF IA from overexpressed subunits reveals stoichiometry and provides insights into molecular topology. Biochemistry. 2011;50:10203–14. 22026644 10.1021/bi200964p PMC3234886

[23] Pancevac C , Goldstone DC , Ramos A , Taylor IA . Structure of the Rna15 RRM – RNA complex reveals the molecular basis of GU specificity in transcriptional 3′‐end processing factors. Nucleic Acids Res. 2010;38:3119–32. 20097654 10.1093/nar/gkq002 PMC2875009

[24] Leeper TC , Qu X , Lu C , Moore C , Varani G . Novel protein–protein contacts facilitate mRNA 3′‐processing signal recognition by Rna15 and Hrp1. J Mol Biol. 2010;401:334–49. 20600122 10.1016/j.jmb.2010.06.032 PMC5512578

[25] Yang FAN , Hsu P , Lee SD , Yang WEN , Hoskinson D , Xu W , et al. The C terminus of Pcf11 forms a novel zinc‐finger structure that plays an essential role in mRNA 3′‐end processing. RNA. 2016;23:98–107. 27780845 10.1261/rna.058354.116 PMC5159653

[26] Guéguéniat J , Dupin AF , Stojko J , Beaurepaire L , Cianf S , Mackereth CD , et al. Distinct roles of Pcf11 zinc‐binding domains in pre‐mRNA 3′‐end processing. Nucleic Acids Res. 2017;45:10115–31. 28973460 10.1093/nar/gkx674 PMC5737669

[27] Takagaki Y , Manley JL , Macdonald CC , Wilusz J , Shenk T . A multisubunit factor, CstF, is required for polyadenylation of mammalian pre‐mRNAs. Genes Dev. 1990;4:2112–20. 1980119 10.1101/gad.4.12a.2112

[28] Yang W , Hsu PL , Yang F , Song J‐E , Varani G . Reconstitution of the CstF complex unveils a regulatory role for CstF‐50 in recognition of 3′‐end processing signals. Nucleic Acids Res. 2017;46:493–503. 10.1093/nar/gkx1177 PMC5778602 29186539

[29] Takagaki Y , Manley JL . RNA recognition by the human polyadenylation factor CstF. Mol Cell Biol. 1997;17:3907–14. 9199325 10.1128/mcb.17.7.3907 PMC232243

[30] de Vries H , Rüegsegger U , Hübner W , Friedlein A , Langen H , Keller W . Human pre‐mRNA cleavage factor II(m) contains homologs of yeast proteins and bridges two other cleavage factors. EMBO J. 2000;19:5895–904. 11060040 10.1093/emboj/19.21.5895 PMC305781

[31] Schäfer P , Tüting C , Schönemann L , Kühn U , Treiber T , Treiber N , et al. Reconstitution of mammalian cleavage factor II involved in 3′ processing of mRNA precursors. RNA. 2018;24:1721–37. 30139799 10.1261/rna.068056.118 PMC6239180

[32] Zhu Y , Wang X , Forouzmand E , Jeong J , Qiao F , Sowd GA , et al. Molecular mechanisms for CFIm‐mediated regulation of mRNA alternative polyadenylation. Mol Cell. 2018;69:62–74.e4. 29276085 10.1016/j.molcel.2017.11.031 PMC5756121

[33] Kim M , Krogan NJ , Vasiljeva L , Rando OJ , Nedea E , Greenblatt JF , et al. The yeast Rat1 exonuclease promotes transcription termination by RNA polymerase II. Nature. 2004;432:517–22. 15565157 10.1038/nature03041

[34] Eaton JD , Davidson L , Bauer DLV , Natsume T , Kanemaki MT , West S . Xrn2 accelerates termination by RNA polymerase II, which is underpinned by CPSF73 activity. Genes Dev. 2018;32:127–39. 29432121 10.1101/gad.308528.117 PMC5830926

[35] Ezeokonkwo C , Zhelkovsky A , Lee R , Bohm A , Moore CL . A flexible linker region in Fip1 is needed for efficient mRNA polyadenylation. RNA. 2011;17:652–64. 21282348 10.1261/rna.2273111 PMC3062176

[36] Kumar A , Yu CWH , Rodríguez‐molina JB , Li X , Freund SMV , Passmore LA . Dynamics in Fip1 regulate eukaryotic mRNA 3′ end processing. Genes Dev. 2021;35:1510–26. 34593603 10.1101/gad.348671.121 PMC8559680

[37] Meinke G , Ezeokonkwo C , Balbo P , Stafford W , Moore C , Bohm A . Structure of yeast poly(A) polymerase in complex with a peptide from Fip1, an intrinsically disordered protein. Biochemistry. 2008;47:6859–69. 18537269 10.1021/bi800204k PMC2615413

[38] Muckenfuss LM , Migenda Herranz AC , Boneberg FM , Clerici M , Jinek M . Fip1 is a multivalent interaction scaffold for processing factors in human mRNA 3′ end biogenesis. Elife. 2022;11:e80332. 36073787 10.7554/eLife.80332 PMC9512404

[39] Hamilton K , Tong L . Molecular mechanism for the interaction between human CPSF30 and hFip1. Genes Dev. 2020;34:1753–61. 33122294 10.1101/gad.343814.120 PMC7706699

[40] Preker PJ , Lingner J , Minvielle‐Sebastia L , Keller W . The FIP1 gene encodes a component of a yeast pre‐mRNA polyadenylation factor that directly interacts with poly(A) polymerase. Cell. 1995;81:379–89. 7736590 10.1016/0092-8674(95)90391-7

[41] He X , Moore C . Regulation of yeast mRNA 3′ end processing by phosphorylation. Mol Cell. 2005;19:619–29. 16137619 10.1016/j.molcel.2005.07.016

[42] Schmid M , Poulsen MB , Olszewski P , Pelechano V , Saguez C , Gupta I , et al. Rrp6p controls mRNA poly(A) tail length and its decoration with poly(A) binding proteins. Mol Cell. 2012;47:267–80. 22683267 10.1016/j.molcel.2012.05.005 PMC3408791

[43] Turtola M , Manav MC , Kumar A , Tudek A , Mroczek S , Krawczyk PS , et al. Three‐layered control of mRNA poly(A) tail synthesis in *Saccharomyces cerevisiae* . Genes Dev. 2021;35:1290–303. 34385261 10.1101/gad.348634.121 PMC8415320

[44] Balbo PB , Bohm A . Mechanism of poly(A) polymerase: structure of the enzyme‐MgATP‐RNA ternary complex and kinetic analysis. Structure. 2007;15:1117–31. 17850751 10.1016/j.str.2007.07.010 PMC2032019

[45] Hopkins DM , Bering T , Bridge L , Bard J , Zhelkovsky AM , Helmling S , et al. Structure of yeast poly(A) polymerase alone and in complex with 3′‐dATP. Science. 2000;289:1346–50. 10958780 10.1126/science.289.5483.1346

[46] Martin G , Keller W . Crystal structure of mammalian poly(A) polymerase in complex with an analog of ATP. EMBO J. 2000;19:4193–203. 10944102 10.1093/emboj/19.16.4193 PMC302044

[47] Balbo PB , Meinke G , Bohm A . Kinetic studies of yeast polyA polymerase indicate an induced fit mechanism for nucleotide specificity. Biochemistry. 2005;44:7777–86. 15909992 10.1021/bi050089r

[48] Lingner J , Radtke I , Wahle E , Keller W . Purification and characterization of poly(A) polymerase from *Saccharomyces cerevisiae* . J Biol Chem. 1991;266:8741–6. 2026590

[49] Wahle E . Purification and characterization of a mammalian polyadenylate polymerase involved in the 3′ end processing of messenger RNA precursors. J Biol Chem. 1991;266:3131–9. 1993684

[50] Martin G , Doublié S , Keller W . Determinants of substrate specificity in RNA‐dependent nucleotidyl transferases. Biochim Biophys Acta. 2008;1779:206–16. 18177750 10.1016/j.bbagrm.2007.12.003 PMC2676681

[51] Bienroth S , Keller W , Wahle E . Assembly of a processive messenger RNA polyadenylation complex. EMBO J. 1993;12:585–94. 8440247 10.1002/j.1460-2075.1993.tb05690.x PMC413241

[52] Kühn U , Gündel M , Knoth A , Kerwitz Y , Rüdel S , Wahle E . Poly(A) tail length is controlled by the nuclear poly(A)‐binding protein regulating the interaction between poly(A) polymerase and the cleavage and polyadenylation specificity factor. J Biol Chem. 2009;284:22803–14. 19509282 10.1074/jbc.M109.018226 PMC2755688

[53] Wahle E . Poly(A) tail length control is caused by termination of processive synthesis. J Biol Chem. 1995;270:2800–8. 7852352 10.1074/jbc.270.6.2800

[54] Minvielle‐Sebastia L , Preker PJ , Keller W . RNA14 and RNA15 proteins as components of a yeast pre‐mRNA 3′‐end processing factor. Science. 1994;266:1702–5. 7992054 10.1126/science.7992054

[55] Venkataraman K , Brown KM , Gilmartin GM . Analysis of a noncanonical poly(A) site reveals a tripartite mechanism for vertebrate poly(A) site recognition. Genes Dev. 2005;19:1315–27. 15937220 10.1101/gad.1298605 PMC1142555

[56] Brown KM , Gilmartin GM . A mechanism for the regulation of pre‐mRNA 3′ processing by human cleavage factor Im. Mol Cell. 2003;12:1467–76. 14690600 10.1016/s1097-2765(03)00453-2

[57] Zhelkovsky AM , Kessler MM , Moore CL . Structure‐function relationships in the *Saccharomyces cerevisiae* poly(A) polymerase. Identification of a novel RNA binding site and a domain that interacts with specificity factor(s). J Biol Chem. 1995;270:26715–20. 7592899 10.1074/jbc.270.44.26715

[58] Zhelkovsky A , Helmling S , Moore C . Processivity of the *Saccharomyces cerevisiae* poly(A) polymerase requires interactions at the carboxyl‐terminal RNA binding domain. Mol Cell Biol. 1998;18:5942–51. 9742111 10.1128/mcb.18.10.5942 PMC109180

[59] Kaufmann I , Martin G . Human Fip1 is a subunit of CPSF that binds to U‐rich RNA elements and stimulates poly(A) polymerase. EMBO J. 2004;23:616–26. 14749727 10.1038/sj.emboj.7600070 PMC1271804

[60] Kerwitz Y , Kühn U , Lilie H , Knoth A , Scheuermann T , Friedrich H , et al. Stimulation of poly(A) polymerase through a direct interaction with the nuclear poly(A) binding protein allosterically regulated by RNA. EMBO J. 2003;22:3705–14. 12853485 10.1093/emboj/cdg347 PMC165617

[61] Eckmann CR , Rammelt C , Wahle E . Control of poly(A) tail length. Wiley Interdiscip Rev RNA. 2011;2:348–61. 21957022 10.1002/wrna.56

[62] Grenier St‐Sauveur V , Soucek S , Corbett AH , Bachand F . Poly(A) tail‐mediated gene regulation by opposing roles of Nab2 and Pab2 nuclear poly(A)‐binding proteins in pre‐mRNA decay. Mol Cell Biol. 2013;33:4718–31. 24081329 10.1128/MCB.00887-13 PMC3838004

[63] Kühn U , Buschmann J , Wahle E . The nuclear poly(A) binding protein of mammals, but not of fission yeast, participates in mRNA polyadenylation. RNA. 2017;23:473–82. 28096519 10.1261/rna.057026.116 PMC5340911

[64] Viphakone N , Voisinet‐Hakil F , Minvielle‐Sebastia L . Molecular dissection of mRNA poly(A) tail length control in yeast. Nucleic Acids Res. 2008;36:2418–33. 18304944 10.1093/nar/gkn080 PMC2367721

[65] Nicholson‐Shaw AL , Kofman ER , Yeo GW , Pasquinelli AE . Nuclear and cytoplasmic poly(A) binding proteins (PABPs) favor distinct transcripts and isoforms. Nucleic Acids Res. 2022;50:4685–702. 35438785 10.1093/nar/gkac263 PMC9071453

[66] Hector RE , Nykamp KR , Dheur S , Anderson JT , Non PJ , Urbinati CR , et al. Dual requirement for yeast hnRNP Nab2p in mRNA poly(A) tail length control and nuclear export. EMBO J. 2002;21:1800–10. 11927564 10.1093/emboj/21.7.1800 PMC125947

[67] Kelly SM , Pabit SA , Kitchen CM , Guo P , Marfatia KA , Murphy TJ , et al. Recognition of polyadenosine RNA by zinc finger proteins. Proc Natl Acad Sci USA. 2007;104:12306–11. 17630287 10.1073/pnas.0701244104 PMC1941466

[68] Fasken MB , Corbett AH , Stewart M . Structure–function relationships in the Nab2 polyadenosine‐RNA binding Zn finger protein family. Protein Sci. 2019;28:513–23. 30578643 10.1002/pro.3565 PMC6371209

[69] Brune C , Munchel SE , Fischer N , Podtelejnikov AV , Weis K . Yeast poly(A)‐binding protein Pab1 shuttles between the nucleus and the cytoplasm and functions in mRNA export. RNA. 2005;11:517–31. 15769879 10.1261/rna.7291205 PMC1370741

[70] Keller RW , Kühn U , Aragón M , Bornikova L , Wahle E , Bear DG . The nuclear poly(A) binding protein, PABP2, forms an oligomeric particle covering the length of the poly(A) tail. J Mol Biol. 2000;297:569–83. 10731412 10.1006/jmbi.2000.3572

[71] Meyer S , Urbanke C , Wahle E . Equilibrium studies on the association of the nuclear poly(A) binding protein with poly(A) of different lengths. Biochemistry. 2002;41:6082–9. 11994003 10.1021/bi0160866

[72] Kühn U , Nemeth A , Meyer S , Wahle E . The RNA binding domains of the nuclear poly(A)‐binding protein. J Biol Chem. 2003;278:16916–25. 12637556 10.1074/jbc.M209886200

[73] Kelly SM , Leung SW , Pak C , Banerjee A , Moberg KH . A conserved role for the zinc finger polyadenosine RNA binding protein, ZC3H14, in control of poly(A) tail length. RNA. 2014;20:681–8. 24671764 10.1261/rna.043984.113 PMC3988569

[74] Aibara S , Gordon JMB , Riesterer AS , Mclaughlin SH , Stewart M . Structural basis for the dimerization of Nab2 generated by RNA binding provides insight into its contribution to both poly(A) tail length determination and transcript compaction in *Saccharomyces cerevisiae* . Nucleic Acids Res. 2017;45:1529–38. 28180315 10.1093/nar/gkw1224 PMC5388407

[75] Baer BW , Kornberg RD . Repeating structure of cytoplasmic poly(A)‐ribonucleoprotein. Proc Natl Acad Sci USA. 1980;77:1890–2. 6929525 10.1073/pnas.77.4.1890 PMC348614

[76] Schäfer IB , Yamashita M , Schuller JM , Schüssler S , Reichelt P , Strauss M , et al. Molecular basis for poly(A) RNP architecture and recognition by the Pan2‐Pan3 deadenylase. Cell. 2019;177:1619–31.e21. 31104843 10.1016/j.cell.2019.04.013 PMC6547884

[77] Subtelny AO , Eichhorn SW , Chen GR , Sive H , Bartel DP . Poly(A)‐tail profiling reveals an embryonic switch in translational control. Nature. 2014;508:66–71. 24476825 10.1038/nature13007 PMC4086860

[78] Jia J , Lu W , Liu B , Fang H , Yu Y , Mo W , et al. An atlas of plant full‐length RNA reveals tissue‐specific and monocots–dicots conserved regulation of poly(A) tail length. Nat Plants. 2021;8:1118–26. 10.1038/s41477-022-01224-9 35982302

[79] Winstall E , Sadowski M , Kühn U , Wahle E , Sachs AB . The *Saccharomyces cerevisiae* RNA‐binding protein Rbp29 functions in cytoplasmic mRNA metabolism. J Biol Chem. 2000;275:21817–26. 10764794 10.1074/jbc.M002412200

[80] Kumar GR , Glaunsinger BA . Nuclear import of cytoplasmic poly(A) binding protein restricts gene expression via hyperadenylation and nuclear retention of mRNA. Mol Cell Biol. 2010;30:4996–5008. 20823266 10.1128/MCB.00600-10 PMC2953054

[81] Hosoda N , Lejeune F , Maquat LE . Evidence that poly(A) binding protein C1 binds nuclear pre‐mRNA poly(A) tails. Mol Cell Biol. 2006;26:3085–97. 16581783 10.1128/MCB.26.8.3085-3097.2006 PMC1446973

[82] Sagawa F , Ibrahim H , Morrison AL , Wilusz CJ , Wilusz J . Nucleophosmin deposition during mRNA 3′end processing influences poly(A) tail length. EMBO J. 2011;30:3994–4005. 21822216 10.1038/emboj.2011.272 PMC3209774

[83] Mitchell SF , Parker R . Principles and properties of eukaryotic mRNPs. Mol Cell. 2014;54:547–58. 24856220 10.1016/j.molcel.2014.04.033

[84] Afonina E , Stauber R , Pavlakis GN . The human poly(A)‐binding protein 1 shuttles between the nucleus and the cytoplasm. J Biol Chem. 1998;273:13015–21. 9582337 10.1074/jbc.273.21.13015

[85] Chen Z , Li Y , Krug RM . Influenza A virus NS1 protein targets poly(A)‐binding protein II of the cellular 3′‐end processing machinery. EMBO J. 1999;18:2273–83. 10205180 10.1093/emboj/18.8.2273 PMC1171310

[86] Burgess HM , Richardson WA , Anderson RC , Salaun C , Graham SV , Gray NK . Nuclear relocalisation of cytoplasmic poly(A)‐binding proteins PABP1 and PABP4 in response to UV irradiation reveals mRNA‐dependent export of metazoan PABPs. J Cell Sci. 2011;124:3344–55. 21940797 10.1242/jcs.087692 PMC3178455

[87] Gilbertson S , Federspiel JD , Hartenian E , Cristea IM , Glaunsinger B . Changes in mRNA abundance drive shuttling of RNA binding proteins, linking cytoplasmic RNA degradation to transcription. Elife. 2018;7:e37663. 30281021 10.7554/eLife.37663 PMC6203436

[88] Carmody SR , Tran EJ , Apponi LH , Corbett AH , Wente SR . The mitogen‐activated protein kinase Slt2 regulates nuclear retention of non‐heat shock mRNAs during heat shock‐induced stress. Mol Cell Biol. 2010;30:5168–79. 20823268 10.1128/MCB.00735-10 PMC2953050

[89] Aguilar L , Paul B , Reiter T , Gendron L , Nambi AAR , Montpetit R , et al. Altered rRNA processing disrupts nuclear RNA homeostasis via competition for the poly(A)‐binding protein Nab2. Nucleic Acids Res. 2020;48:11675–94. 33137177 10.1093/nar/gkaa964 PMC7672433

[90] Oeffinger M , Zenklusen D . To the pore and through the pore: a story of mRNA export kinetics. Biochim Biophys Acta. 2012;1819:494–506. 22387213 10.1016/j.bbagrm.2012.02.011 PMC3345096

[91] Qu X , Lykke‐Andersen S , Nasser T , Saguez C , Bertrand E , Jensen TH , et al. Assembly of an export‐competent mRNP is needed for efficient release of the 3′‐end processing complex after polyadenylation. Mol Cell Biol. 2009;29:5327–38. 19635808 10.1128/MCB.00468-09 PMC2747973

[92] Rigo F , Martinson HG . Polyadenylation releases mRNA from RNA polymerase II in a process that is licensed by splicing. RNA. 2009;15:823–36. 19304926 10.1261/rna.1409209 PMC2673064

[93] Stewart M . Polyadenylation and nuclear export of mRNAs. J Biol Chem. 2019;294:2977–87. 30683695 10.1074/jbc.REV118.005594 PMC6398137

[94] Tudek A , Lloret‐Llinares M , Jensen TH . The multitasking polyA tail: nuclear RNA maturation, degradation and export. Philos Trans R Soc Lond B Biol Sci. 2018;373:20180169. 30397105 10.1098/rstb.2018.0169 PMC6232593

[95] Kamieniarz‐Gdula K , Proudfoot NJ . Transcriptional control by premature termination: a forgotten mechanism. Trends Genet. 2019;35:553–64. 31213387 10.1016/j.tig.2019.05.005 PMC7471841

[96] Venters CC , Oh J , Di C , So BR , Dreyfuss G . U1 snRNP telescripting: suppression of premature transcription termination in introns as a new layer of gene regulation. Cold Spring Harb Perspect Biol. 2019;11:a032235. 30709878 10.1101/cshperspect.a032235 PMC6360859

[97] Rouvière JO , Lykke‐Andersen S , Jensen TH . Control of non‐productive RNA polymerase II transcription via its early termination in metazoans. Biochem Soc Trans. 2022;50:283–95. 35166324 10.1042/BST20201140

[98] Mitschka S , Mayr C . Context‐specific regulation and function of mRNA alternative polyadenylation. Nat Rev Mol Cell Biol. 2022;23:779–96. 35798852 10.1038/s41580-022-00507-5 PMC9261900

[99] Geisberg JV , Moqtaderi Z , Struhl K . The transcriptional elongation rate regulates alternative polyadenylation in yeast. Elife. 2020;9:e59810. 32845240 10.7554/eLife.59810 PMC7532003

[100] Ozsolak F , Kapranov P , Foissac S , Kim SW , Fishilevich E , Monaghan AP , et al. Comprehensive polyadenylation site maps in yeast and human reveal pervasive alternative polyadenylation. Cell. 2010;143:1018–29. 21145465 10.1016/j.cell.2010.11.020 PMC3022516

[101] Colgan DF , Murthy KGK , Prives C , Manley JL . Cell‐cycle related regulation of poly(A) polymerase by phosphorylation. Nature. 1996;384:282–5. 8918882 10.1038/384282a0

[102] Mizrahi N , Moore C . Posttranslational phosphorylation and ubiquitination of the *Saccharomyces cerevisiae* poly(A) polymerase at the S/G2 stage of the cell cycle. Mol Cell Biol. 2000;20:2794–802. 10733582 10.1128/mcb.20.8.2794-2802.2000 PMC85495

[103] Kim H , Lee JH , Lee Y . Regulation of poly(A) polymerase by 14‐3‐3ɛ. EMBO J. 2003;22:5208–19. 14517258 10.1093/emboj/cdg486 PMC204469

[104] Saguez C , Schmid M , Olesen JR , Ghazy MAEH , Qu X , Poulsen MB , et al. Nuclear mRNA surveillance in THO/sub2 mutants is triggered by inefficient polyadenylation. Mol Cell. 2008;31:91–103. 18614048 10.1016/j.molcel.2008.04.030

[105] Vethantham V , Rao N , Manley JL . Sumoylation regulates multiple aspects of mammalian poly(A) polymerase function. Genes Dev. 2008;22:499–511. 18281463 10.1101/gad.1628208 PMC2238671

[106] Di Giammartino DC , Shi Y , Manley JL . Article PARP1 represses PAP and inhibits polyadenylation during heat shock. Mol Cell. 2013;49:7–17. 23219533 10.1016/j.molcel.2012.11.005 PMC3545032

[107] Shimazu T , Horinouchi S , Yoshida M . Multiple histone deacetylases and the CREB‐binding protein regulate pre‐mRNA 3′‐end processing. J Biol Chem. 2007;282:4470–8. 17172643 10.1074/jbc.M609745200

[108] Lutz CS , Murthy KGK , Schek N , Connor JPO , Manley JL , Alwine JC . Interaction between the U1 snRNP‐A factor increases polyadenylation efficiency in vitro. Genes Dev. 1996;10:325–37. 8595883 10.1101/gad.10.3.325

[109] Gunderson SI , Vagner S , Polycarpou‐schwarz M , Mattaj IW . Involvement of the carboxyl terminus of vertebrate poly(A) polymerase in U1A autoregulation and in the coupling of splicing and polyadenylation. Genes Dev. 1997;11:761–73. 9087430 10.1101/gad.11.6.761

[110] Gunderson SI , Polycarpou‐schwarz M , Mattaj IW . U1 snRNP inhibits pre‐mRNA polyadenylation through a direct interaction between U1 70K and poly(A) polymerase. Mol Cell. 1998;1:255–64. 9659922 10.1016/s1097-2765(00)80026-x

[111] Ko B , Gunderson SI . Identification of new poly(A) polymerase‐inhibitory proteins capable of regulating pre‐mRNA polyadenylation. J Mol Biol. 2002;318:1189–206. 12083511 10.1016/s0022-2836(02)00240-1

[112] Kyburz A , Friedlein A , Langen H , Keller W . Direct interactions between subunits of CPSF and the U2 snRNP contribute to the coupling of pre‐mRNA 3′ end processing and splicing. Mol Cell. 2006;23:195–205. 16857586 10.1016/j.molcel.2006.05.037

[113] Danckwardt S , Kaufmann I , Gentzel M , Foerstner KU , Gantzert A , Gehring NH , et al. Splicing factors stimulate polyadenylation via USEs at non‐canonical 3′ end formation signals. EMBO J. 2007;26:2658–69. 17464285 10.1038/sj.emboj.7601699 PMC1888663

[114] Niwa M , Rose SD , Berget SM . In vitro polyadenylation is stimulated by the presence of an upstream intron. Genes Dev. 1990;4:1552–9. 1701407 10.1101/gad.4.9.1552

[115] Davidson L , West S . Splicing‐coupled 3′end formation requires a terminal splice acceptor site, but not intron excision. Nucleic Acids Res. 2013;41:7101–14. 23716637 10.1093/nar/gkt446 PMC3737548

[116] Reimer KA , Mimoso CA , Adelman K , Neugebauer KM , Reimer KA , Mimoso CA , et al. Co‐transcriptional splicing regulates 3′end cleavage during mammalian erythropoiesis. Mol Cell. 2021;81:998–1012.e7. 33440169 10.1016/j.molcel.2020.12.018 PMC8038867

[117] Vagner C , Mattaj IW . The carboxyl terminus of vertebrate poly(A) polymerase interacts with U2AF 65 to couple 3′end processing and splicing. Genes Dev. 2000;14:403–13. 10691733 PMC316384

[118] Muniz L , Davidson L , West S . Poly(A) polymerase and the nuclear poly(A) binding protein, PABPN1, coordinate the splicing and degradation of a subset of human pre‐mRNAs. Mol Cell Biol. 2015;35:2218–30. 25896913 10.1128/MCB.00123-15 PMC4456446

[119] Jumper J , Evans R , Pritzel A , Green T , Figurnov M , Ronneberger O , et al. Highly accurate protein structure prediction with AlphaFold. Nature. 2021;596:583–9. 34265844 10.1038/s41586-021-03819-2 PMC8371605

[120] Varadi M , Anyango S , Deshpande M , Nair S , Natassia C , Yordanova G , et al. AlphaFold protein structure database: massively expanding the structural coverage of protein‐sequence space with high‐accuracy models. Nucleic Acids Res. 2022;50:D439–44. 34791371 10.1093/nar/gkab1061 PMC8728224

[121] Dower K , Kuperwasser N , Merrikh H , Rosbash M . A synthetic A tail rescues yeast nuclear accumulation of a ribozyme‐terminated transcript. RNA. 2004;10:1888–99. 15547135 10.1261/rna.7166704 PMC1370677

[122] Schmid M , Jensen TH . Controlling nuclear RNA levels. Nat Rev Genet. 2018;19:518–29. 29748575 10.1038/s41576-018-0013-2

[123] Dominski Z , Tong L . U7 deciphered: the mechanism that forms the unusual 3′ end of metazoan replication‐dependent histone mRNAs. Biochem Soc Trans. 2021;49:2229–40. 34351387 10.1042/BST20210323 PMC8563397

[124] Xiang K , Bartel DP . The molecular basis of coupling between poly(A)‐tail length and translational efficiency. Elife. 2021;10:e66493. 34213414 10.7554/eLife.66493 PMC8253595

[125] Passmore LA , Coller J . Roles of mRNA poly(A) tails in regulation of eukaryotic gene expression. Nat Rev Mol Cell Biol. 2021;23:93–106. 34594027 10.1038/s41580-021-00417-y PMC7614307

[126] Parker R . RNA degradation in *Saccharomyces cerevisae* . Genetics. 2012;191:671–702. 22785621 10.1534/genetics.111.137265 PMC3389967

[127] Webster MW , Chen YH , Stowell JAW , Alhusaini N , Sweet T , Graveley BR , et al. mRNA deadenylation is coupled to translation rates by the differential activities of Ccr4‐not nucleases. Mol Cell. 2018;70:1089–100. 29932902 10.1016/j.molcel.2018.05.033 PMC6024076

[128] Yi H , Park J , Ha M , Lim J , Chang H , Kim VN . PABP cooperates with the CCR4‐NOT complex to promote mRNA deadenylation and block precocious decay. Mol Cell. 2018;70:1081–8. 29932901 10.1016/j.molcel.2018.05.009

[129] Chang H , Lim J , Ha M , Kim VN . TAIL‐seq: genome‐wide determination of poly(A) tail length and 3′ end modifications. Mol Cell. 2014;53:1044–52. 24582499 10.1016/j.molcel.2014.02.007

[130] Eisen TJ , Eichhorn SW , Subtelny AO , Lin KS , McGeary SE , Gupta S , et al. The dynamics of cytoplasmic mRNA metabolism. Mol Cell. 2020;77:786–99.e10. 31902669 10.1016/j.molcel.2019.12.005 PMC7265681

[131] Tudek A , Krawczyk PS , Mroczek S , Tomecki R , Turtola M , Matylla‐Kulińska K , et al. Global view on the metabolism of RNA poly(A) tails in yeast *Saccharomyces cerevisiae* . Nat Commun. 2021;12:4951. 34400637 10.1038/s41467-021-25251-w PMC8367983

[132] Azoubel Lima S , Chipman LB , Nicholson AL , Chen YH , Yee BA , Yeo GW , et al. Short poly(A) tails are a conserved feature of highly expressed genes. Nat Struct Mol Biol. 2017;24:1057–63. 29106412 10.1038/nsmb.3499 PMC5877826

[133] Wiener D , Antebi Y , Schwartz S . Decoupling of degradation from deadenylation reshapes poly(A) tail length in yeast meiosis. Nat Struct Mol Biol. 2021;28:1038–49. 34887567 10.1038/s41594-021-00694-3

[134] Charlesworth A , Meijer HA , de Moor CH . Specificity factors in cytoplasmic polyadenylation. Wiley Interdiscip Rev RNA. 2013;4:437–61. 23776146 10.1002/wrna.1171 PMC3736149

[135] Liudkovska V , Dziembowski A . Functions and mechanisms of RNA tailing by metazoan terminal nucleotidyltransferases. Wiley Interdiscip Rev RNA. 2021;12:e1622. 33145994 10.1002/wrna.1622 PMC7988573

[136] Lim J , Kim D , Lee Y , Ha M , Lee M , Yeo J , et al. Mixed tailing by TENT4A and TENT4B shields mRNA from rapid deadenylation. Science. 2018;361:701–4. 30026317 10.1126/science.aam5794

[137] Viegas IJ , de Macedo JP , Serra L , De Niz M , Temporão A , Pereira SS , et al. N6‐methyladenosine in poly(A) tails stabilize VSG transcripts. Nature. 2022;604:362–70. 35355019 10.1038/s41586-022-04544-0 PMC9150445

[138] Hunt AG , Xing D , Li QQ . Plant polyadenylation factors: conservation and variety in the polyadenylation complex in plants. BMC Genomics. 2012;13:641. 23167306 10.1186/1471-2164-13-641 PMC3538716

